# Intravoxel incoherent motion magnetic resonance imaging findings in the acute phase of MELAS: a case report

**DOI:** 10.1002/brb3.282

**Published:** 2014-09-11

**Authors:** Ryuji Uehara, Koji Yamashita, Akio Hiwatashi, Osamu Togao, Kazufumi Kikuchi, Jun Yokoyama, Dai Matsuse, Takashi Yoshiura, Hiroshi Honda

**Affiliations:** 1 Department of Clinical Radiology, Graduate School of Medical Sciences, Kyushu University3-1-1, Maidashi, Higashi-ku, Fukuoka, 812-8582, Japan; 2 Department of Neurology, Graduate School of Medical Sciences, Kyushu University3-1-1, Maidashi, Higashi-ku, Fukuoka, 812-8582, Japan

**Keywords:** Diffusion MRI, IVIM, MELAS, perfusion MRI

## Abstract

**Objective:**

We report the clinical application of intravoxel incoherent motion (IVIM) magnetic resonance (MR) imaging to diagnose a case of mitochondrial myopathy, encephalopathy, lactic acidosis, and stroke-like episodes (MELAS) in the acute phase.

**Results:**

On IVIM MR Images of this patient, higher perfusion (f) and diffusion (D) values in the left occipital and temporal lobes were found compared to the contralateral areas.

**Conclusion:**

These findings imply a breakdown of autoregulation with hyperperfusion and vasogenic edema during the acute phase of MELAS, as described in previous reports. IVIM imaging is a valuable, noninvasive tool that simultaneously quantifies perfusion and diffusion parameters.

## Introduction

Stroke-like episodes are the most common symptoms of mitochondrial myopathy, encephalopathy, lactic acidosis, and stroke-like episodes (MELAS). MELAS is physiologically characterized by hyperperfusion and vasogenic edema in the acute phase (Iizuka et al. 2007). The radiological findings of MELAS have been well documented. The increase in focal perfusion has been reported using single-photon emission computed tomography (Nishioka et al. 2008), dynamic susceptibility contrast perfusion magnetic resonance (MR) imaging, and arterial spin labeling (ASL) (Takasu et al. 2002; Tsujikawa et al. 2010). In addition, an increase in the apparent diffusion coefficient has been observed in previous studies (Yoneda et al. 1999; Kolb et al. 2003). Intravoxel incoherent motion (IVIM) MR imaging is a noninvasive technique that simultaneously determines the perfusion fraction (f) and diffusion coefficient (D) (Le Bihan et al. 1988). We report a patient in the acute phase of MELAS who showed characteristic radiological findings with IVIM imaging.

## Case report

A 24-year-old male was admitted to our institution with complaints of headaches and right homonymous hemianopsia. He developed sensorineural hearing loss at the age of 18 years. Five months prior to admission, he was diagnosed with MELAS based on a point mutation in the mitochondrial A3243G gene. He experienced a second seizure 9 days after an appendectomy. Although the seizures were well controlled with anticonvulsants, 3 weeks later, he again reported headaches and right homonymous hemianopsia. The concentrations of lactate and pyruvate in his blood on admission were 28.3 and 1.61 mg/dL (normal range: 4.7–18.7 and 0.30–0.94 mg/dL), respectively. MR images were obtained with a 3.0-T MR unit (Achieva 3.0 T TX, Philips Medical Systems, Best, Netherlands). Conventional MR imaging, ASL (TR/TE = 4200/8.6 ms, postlabeling delay = 1525 ms, 64 × 64 matrix, NEX = 1, slice thickness = 6 mm), and IVIM imaging were obtained. Conventional MR imaging included T2-weighted imaging (T2WI; TR/TE = 3000/80 ms), FLAIR (TR/TE/TI = 10,000/120/2700 ms) and diffusion-weighted imaging (DWI; TR/TE = 3421/62 ms, b value = 1000 s/mm^2^). IVIM imaging (TR/TE = 2500/70 ms) was performed with 13 different b values (0, 10, 20, 30, 50, 80, 100, 200, 300, 400, 600, 800, 1000 s/mm^2^) in three orthogonal directions. Data were analyzed with Philips Research Integrated Development Environment software written in Interactive Data Language 6.3 (ITT Visual Information Solutions, Boulder, CO; Pang et al. 2013).

**Figure 1:**
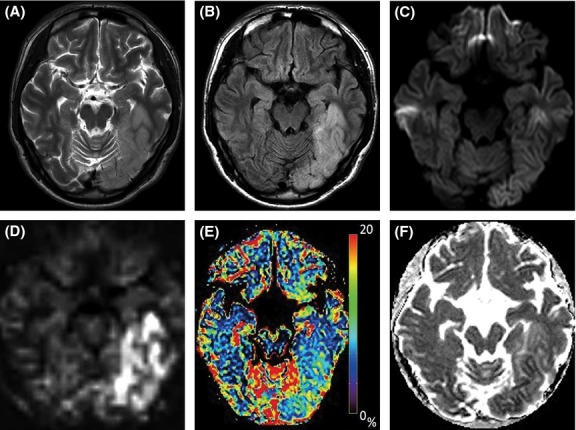
Transverse magnetic resonance (MR) images. T2WI (A) and FLAIR image (B) show hyperintensity in the left temporo-occipital lobe. diffusion-weighted imaging (DWI) (C) demonstrates mild hyperintensity in this area. arterial spin labeling (ASL) image (D) reveals hyperintensity, which indicates hyperperfusion in this area. (E) f map and (F) D map from intravoxel incoherent motion (IVIM) image. Both the mean f and D values in the left temporo-occipital lobe lesion (8.2% and 0.92 × 10^−3 ^mm^2^/s, respectively) are higher than in the contralateral area (4.3% and 0.71 × 10^−3 ^mm^2^/s, respectively).

T2WI and FLAIR showed hyperintensity in the left temporo-occipital lobe. DWI demonstrated slight hyperintensity in this area. ASL imaging revealed hyperintensity, which indicated hyperperfusion in this area. With IVIM imaging, the mean f and D values in the left temporo-occipital lobe lesion (8.2% and 0.92 × 10^−3 ^mm^2^/s, respectively) were higher than in the contralateral area (4.3% and 0.71 × 10^−3 ^mm^2^/s, respectively; Fig.1). The patient was treated with oral carbamazepine followed by l-arginine. The clinical course was favorable, and no increase in his symptoms was seen. At 3-week follow-up, regression of the left temporo-occipital lesion was seen on IVIM images (not shown). The patient was discharged after 5 weeks in the hospital.

## Discussion

Intravoxel incoherent motion imaging was introduced in 1986 by Le Bihan et al. (1986) to separate the signal into a diffusion and perfusion components with different exponential decays. IVIM imaging parameters were calculated with biexponential fitting to the equation: 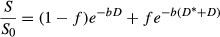
where *S* is the signal intensity, *S*_*0*_ is the signal intensity when the *b* value was 0 sec/mm^2^, *b* is the diffusion-weighting factor, *f* is the perfusion fraction, *D* is the diffusion coefficient, and D* is pseudodiffusion coefficient. IVIM imaging provides promising data and has been reported to be useful for assessing brain tumors, head and neck tumors, and breast cancer (Sigmund et al. 2011; Sumi et al. 2012; Federau et al. 2014).

MELAS is a disorder of adenosine triphosphate (ATP) production due to a point mutation of mitochondrial DNA (Moudy et al. 1995). Stroke-like episodes are the most common clinical features of MELAS and are characterized by increased capillary permeability, hyperperfusion, neuronal vulnerability, and neuronal hyperexcitability (Moudy et al. 1995; Iizuka et al. 2007). The pathophysiology during acute phase of MELAS remains unknown. One hypothesis is that an energy imbalance exists between energy requirement and the availability of ATP, which is due to relatively insufficient oxidative phosphorylation, and that this imbalance causes exaggerated anaerobic glycolysis and lactic acidosis (Iizuka et al. 2007). Lactic acidosis in the surrounding area of the acute brain lesions may decrease the pH in the smooth muscle cells of the vessels, leading to vasodilatation and hyperemia (Iizuka et al. 2007; Kim et al. 2011). As a result, the lesions show hyperperfusion and vasogenic edema during the active phase of MELAS. Our results suggest that increased f and D values are due to hyperperfusion and vasogenic edema, respectively. In addition, the IVIM imaging findings and clinical course of the patient show the potential of IVIM imaging to predict the treatment response.

Although it is difficult to eliminate the possibility of the post-seizure or ongoing seizure effects, genetic diagnosis with MELAS has been already achieved.

In conclusion, we report a patient in the acute phase of MELAS who appears to show characteristic radiological findings using IVIM imaging. This type of imaging is a valuable technique that allows noninvasive and simultaneous measurement of both regional perfusion and diffusion.
